# Implementation of Physical Activity into routine Clinical pracTice in Rheumatic Musculoskeletal Disease: The IMPACT-RMD study protocol and rationale

**DOI:** 10.31138/mjr.30.4.231

**Published:** 2019-12-31

**Authors:** George S. Metsios, Sally AM Fenton, Helene Rikke Moe, Martin van der Esch, Jet Veldhuijzen van Zanten, Yannis Koutedakis, Panagiotis Vitalis, Norelee Kennedy, Nina C. Brodin, Aikaterini Tzika, Carina Boström, Thijs Willem Swinnen, Rebecca Jester, Karin Niedermann Schneider, Elena Nikiphorou, George E. Fragoulis, Thea P.M. Vliet Vlieland, Cornelia Van den Ende, George D. Kitas, The IMPACT-RMD Consortium.

**Affiliations:** 1 Faculty of Education, Health and Wellbeing, University of Wolverhampton, United Kingdom,; 2 Department of Rheumatology, Russells Hall Hospital, Dudley Group NHS Foundation Trust, Dudley, United Kingdom,; 3 School of Physical Education and Sport Science, University of Thessaly, Greece,; 4 School of Sport, Exercise and Rehabilitation Sciences, University of Birmingham, Birmingham, United Kingdom,; 5 National Resource Centre for Rehabilitation in Rheumatology, Department of Rheumatology, Diakonhjemmet Hospital, Oslo, Norway,; 6 ACHIEVE - Center of Applied Research, Faculty of Health, Amsterdam University of Applied Sciences. Reade, Center for Rehabilitation and Rheumatology/Amsterdam Rehabilitation Research Center, Amsterdam, The Netherlands,; 7 School of Allied Health, Faculty of Education and Health Sciences and Health Research Institute, University of Limerick, Limerick, Ireland,; 8 Department of Neurobiology, Care Sciences and Society, Division of Physiotherapy, Karolinska Institutet, Huddinge, Sweden,; 9 Division of Rheumatology, University Hospitals Leuven, Leuven, Belgium,; 10 School of Health Professions, Institute of Physiotherapy, Zurich University of Applied Sciences, Winterthur, Switzerland,; 11 School of Immunology and Microbial Sciences, King’s College, London, UK,; 12 Institute of Infection, Immunity and Inflammation, University of Glasgow, Glasgow, UK,; 13 Department of Orthopaedics, Rehabilitation and Physical Therapy, Leiden University Medical Center, Leiden, the Netherlands,; 14 Department of Rheumatology, Sint Maartenskliniek, Nijmegen, the Netherlands

**Keywords:** physical activity, exercise, implementation, e-Learning, education

## Abstract

**Background:**

Physical activity is an important intervention for improving disease-related symptoms and systemic manifestations in rheumatic and musculoskeletal disease (RMDs). However, studies suggest that RMD patients report that the lack of individualized and consistent information about physical activity from managing doctors and healthcare professionals, acts as a barrier for engagement. On the other hand, managing doctors and healthcare professionals report lack of knowledge in this area and thus lack of confidence to educate and advise RMD patients about the beneficial effects of physical activity. The aim of the present study therefore, is to develop two e-Learning courses for RMD doctors and health professionals: a) the first one to provide consistent information about the collective benefits of physical activity in RMDs and b) the second on how to implement physical activity advice in routine clinical practice.

**Methods:**

An international collaboration of seven countries, consisting of one academic institution and one patient organization from each country, will co-develop the two e-Learning courses. The final e-Learning courses will primarily target to improve – through physical activity advice – RMD symptoms which are important for patients.

**Discussion:**

The main result of this study will be to co-develop two e-Learning courses that can be used by managing RMD doctors and healthcare professionals to be made aware of the overall benefits of physical activity in RMDs as well as how to implement physical activity advise within their practice.

## INTRODUCTION

Rheumatic and musculoskeletal diseases (RMDs) represent a group of more than 200 diverse non-communicable diseases, that affect both children and adults. There are common symptoms that characterize all RMDs, and it is these manifestations alongside common patho-physiological pathways that have led to the grouping of these conditions under the umbrella term “RMDs”. In general, the symptoms characterising RMDs are pain, fatigue, and joint damage, and subsequently loss of range of motion and function in one or more areas of the musculoskeletal system. The European League Against Rheumatism (EULAR) estimates that RMDs affect one quarter of the European population, with an estimated EU healthcare cost at 2% of its gross domestic product (GDP).^1^ As such, identifying strategies that may alleviate this significant burden, both for the individual as well as the society, is and should be a strong point of public health and healthcare focus.

A promising and safe intervention that can significantly contribute to better management of RMDs is physical activity. Accumulated evidence from systematic reviews and meta-analyses demonstrates that increasing physical activity and/or exercise (ie, structured and planned physical activity) may significantly improve both patient- and clinically-important outcomes in RMDs.^2–4^ Notably, the Cochrane Collaboration has published a significant amount of meta-analyses on the effects of exercise on different outcomes in different RMDs, such as the beneficial effects of exercise on physical function and pain in rheumatoid arthritis (RA) and lower-limb osteoarthritis.^2,5^ Other systematic reviews report consistent findings, demonstrating beneficial effects of physical activity and exercise on various different disease-related outcomes (such as fatigue) as well as systemic manifestations in RMDs (such as cardiovascular disease risk and cachexia).^6–10^

Still, despite the published evidence for the multiple benefits of physical activity and/or cardiorespiratory fitness in RMDs, observational studies consistently demonstrate that physical activity and cardiorespiratory fitness levels are lower in RMD patients compared to the general population.^11–15^ The debilitating symptoms of RMDs feature as predominant reasons for the observed lack of physical activity engagement in this patient population.^16,17^ Specifically, fear of aggravating symptoms, joint damage, pain and fatigue are frequently reported barriers to physical activity. However, it is important to note that it is these symptoms that tend to improve as a result of engaging with physical activity, while patients report they are aware of these benefits.^2,5^

Alongside these individual-level disease-related barriers, barriers to physical activity engagement also operate at the organizational-level for RMD patients, and specifically, within the healthcare system. In specific, lack of provision of physical activity information in routine clinical practice, inconsistency in the information provided by frontline healthcare staff (eg, doctors, nurses, physiotherapists), as well as lack of RMD specific physical activity programs and knowledgeable/skilled exercise instructors, have all been highlighted by RMD patients as significant barriers for engaging with physical activity and/or exercise.^16–18^

These issues can be largely attributed to a lack of evidence-based education and training for RMD health professionals. As a result, implementation of physical activity is always relying on individual efforts to encourage physical activity among RMD patients and/or research funding to develop, deliver and evaluate short-term interventions, for which any positive changes observed in behaviour are not maintained following the cessation of the program. Thus, current efforts to promote more physical activity and exercise among RMD patients represent unsustainable solutions to the problem of physical inactivity in RMD.

So, how is it possible to consistently implement effective physical activity interventions for RMDs within the healthcare system, bearing in mind all these significant barriers? Implementation science suggests that implementation of successful healthcare interventions can be achieved either via legislation or disruptive social innovation.^19^ Given the lack of relevant legislation, ie, national healthcare systems funding physical activity rehabilitation in RMDs, implementation research studies have no option but to currently focus on disruptive social innovation. To achieve this, all key implementers in the implementation chain (eg, patients, managing doctors and healthcare professionals), should contribute to delivery of the physical activity interventions. Based on the current state-of-the-art in RMDs, this should initiate within routine clinical practice as the first point of contact. This is corroborated by research findings and anecdotal evidence, suggesting that RMD patients require their trusted managing healthcare professionals to act as a “trigger” that will help them change their behaviours and become more physically active.^16,17^

However, in the case of RMDs, the medical and healthcare professional curricula do not consistently incorporate dedicated modules that describe the effects of physical activity on RMD symptoms or how to incorporate physical activity in routine clinical practice to better manage symptoms of RMDs. This knowledge is critical to ensure healthcare professionals feel competent to engage in conversations about management of RMDs through physical activity with their patients, and thus enhance the adoption of such approaches. In other non-communicable diseases, when clinicians in primary care were trained to deliver brief physical activity interventions during routine patient visits, this resulted in increased levels of physical activity (2-year follow-up) and significant improvements in cardiorespiratory fitness.^20^ Recent trials^21,22^ also provided strong evidence that brief lifestyle/physical activity counselling among adults with prehypertension and hypertension and/or diabetes resulted in a significant reduction in CVD risk with excellent sustainability. This suggests that adequate expertise and brief advice have the potential to achieve such beneficial changes. However, no such studies exist for RMDs.

The present study, therefore, aims to co-develop together with RMD patients (ie, RMD patients identifying the outcomes to be targeted) two distinct e-Learning courses that address two salient barriers to promoting physical activity participation: a) knowledge of the beneficial effects of physical activity for RMD symptoms, and b) how to deliver consistent physical activity advice during routine clinical visits. These courses will be developed by the IMPACT-RMD consortium, comprised of academic partners and patient organizations from seven countries across the EU and EULAR, and will serve as a critical starting point to implementing physical activity in RMD clinical practice.

## METHODS

### Development of two e-Learning courses

*Workgroup Composition:* This study is a collaboration of both research entities and patient organizations from the following seven participating countries, specifically:

1. UK: Universities of Wolverhampton and Birmingham and the National Rheumatoid Arthritis Association,
2. Ireland: University of Limerick and the Arthritis Ireland,
3. Netherlands: Reade, Centre for Rehabilitation and Rheumatology Amsterdam University of Applied Sciences and the Dutch Arthritis Association,
4. Switzerland: the Zurich University of Applied Sciences, represented by the convenor of the current 2018 EULAR recommendations for physical activity (4) and the RA Patient Organization,
5. Sweden: the Karolinska Institute and the Swedish Rheumatism Association,
6. Belgium: the University Hospital Leuven and RheumaNet, represented by the Chair of the Standing Committee of the EULAR PARE, and
7. Greece: University of Thessaly and the Hellenic League Against Rheumatism.

The project is also actively supported by two EMEUNET EULAR and two PARE members throughout its development and implementation, as well as the present Chair of: a) the EULAR Health Professionals in Rheumatology (HPR) and b) the EULAR Physical Activity and Exercise Therapy Study Group and finally, members of the EULAR Educational Team.

Together, the IMPACT-RMD consortium will develop e-Learning courses via a 3-step process.

Step 1: Understand implementation barriers from both RMD patients as well as frontline healthcare staff.

As per the relevant implementation framework provided by the World Health Organization (WHO),^23^ the present methods will help understand implementation barriers perceived by individuals at both ends of the implementation continuum: those that deliver the intervention (front-line healthcare staff), and those receiving the intervention (RMD patients). The concept of the whole project is to deliver the information in a patient-centred manner, ie, to predominantly address outcomes identified by patients. As such, Step 1 will seek to; a) understand patient-important outcomes, and b) identify implementation barriers in clinical practice across the participating countries.

*Understand patient-important outcomes*: The e-Learning courses aim to equip RMD healthcare professionals with the knowledge and skills to be able to deliver physical activity advice to patients in order to improve outcomes, that patients themselves perceive are the most important. As such, three distinct approaches will be utilized to identify these specific patient-important outcomes:

- a literature review of studies investigating patient-important barriers for engaging in physical activity will be conducted. This will consist of systematic reviews, as well as individual qualitative and quantitative studies,
- one representative from each patient organization per collaborating country will be asked to fill in a short questionnaire to rank identified symptoms, based on how important they are perceived for patients, and
- to provide more depth and dearth of data, the same short questionnaire will be distributed via the EULAR PARE platform to individual patients with RMDs, within the IMPACT-RMD participating countries.

**Figure 1.**
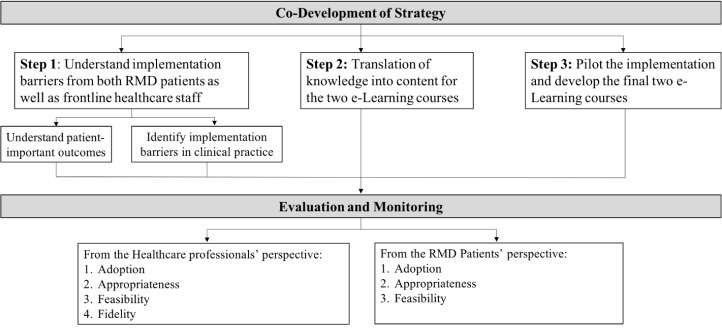
Step-by-step and informed process of developing the e-Learning Courses.

Data will be triangulated to inform the content and focus of e-Learning resources, to ensure healthcare practitioners are addressing the most salient barriers to physical activity participation in routine patient consultations.

*Identifying implementation barriers in clinical practice across European countries:* IMPACT-RMD collaborators in all participating countries will undertake group interviews and focus groups with RMD health professionals. The aim of these discussions will be to establish current (country-specific) physical activity implementation barriers for advising RMD patients to engage in physical activity. Understanding in more depth the current implementation barriers that exist within each country’s healthcare system, will help to inform the development of an “implementation map” in every participating country. Relevant literature findings from systematic reviews and qualitative and quantitative studies on physical activity in RMDs will also inform this step.

Step 2: Translation of knowledge into content for the two e-Learning courses.

The above resources will be discussed in an Expert Review meeting with all collaborating IMPACT-RMD partners, including patient organizations, researchers involved in the development of the 2018 EULAR physical activity guidelines^4^ as well as the current Chairs of the PARE, EULAR HPRs, the EULAR Physical Activity and Exercise study group and the EULAR non-Pharmacological Treatment of Autoimmune Connective Tissue Diseases study group. The end result of this meeting will be to solidify the main patient-important outcomes that will be addressed in the two e-Learning courses. Using the interactive educational platforms, the EULAR Educational Team will provide the means of developing the two e-Learning courses.

Step 3: Pilot the implementation and developing the final two e-Learning courses.

After the development of the two e-Learning courses, each of the collaborators from IMPACT-RMD countries will introduce and implement the e-Learning courses in their respective hospitals. Healthcare professionals in these hospitals will be asked to undertake the e-Learning courses to: a) understand the overall effects of physical activity on disease-related outcomes and systemic manifestations, and b) learn how to implement consistent physical activity advise during routine clinical practice based on the RMD patients’ needs, preferences and functional ability. Feedback, through short face-to-face interviews, will be provided by those healthcare professionals who completed the course and provided information/advice to RMD patients but also from the patients receiving the physical activity advice.

As per the utilized implementation framework from WHO,^23^ this stage will gather qualitative data that will address implementation barriers and facilitators for both healthcare professionals and RMD patients. This information will be then be used to develop the content for the two final e-Learning courses.

*Doctors and Healthcare professionals:* The face-to-face interviews will evaluate:

-Adoption (did they adhere to the e-Learning physical activity advise suggestions),
-Appropriateness (was it relevant to the gaps in the doctors and healthcare professionals’ knowledge and evidence-based practice),
-Feasibility (could they deliver the physical activity information practically and can this be used from now on in their everyday practice),
-Fidelity (did the learning courses help provide individualized physical activity advise).

*Patients*: the face-to-face interviews with RMD patients after receiving the physical activity advice will evaluate:

-Adoption (did the physical activity advice enable the uptake of physical activity),
-Appropriateness (was acceptability and suitability perceived, and did the information address important-patient outcomes),
-Feasibility (was the information provided in a practical way).

## DISCUSSION

This study has adopted an implementation framework approach, developed by the WHO,^23^ in order to co-develop – in a step-by-step informed process – two e-Learning courses that intend to support the implementation of physical activity advise within routine clinical practice. The choice to develop e-Learning material, rather than any other educational approaches (eg, webinars, traditionally taught courses), is underpinned by relevant evidence and preliminary focus group work. In specific, collective literature findings reveal that e-Learning is equally effective as traditional education for healthcare professionals^24^ and that e-Learning can also be a way of continuous and sustainable professional development.^25^ This is also supported by research in RMDs: RMD healthcare professionals prefer online interactive courses for their continuous personal development.^26^ The two IMPACT-RMD e-Learning courses aim to address important barriers identified in the literature (for both RMD patients and treating healthcare professionals). Currently, no such e-learning courses exist for RMD professionals, in particular, courses that directly address patient-important outcomes. However, in other populations with non-communicable diseases, physical activity advice during routine clinical care provided strong evidence that brief counselling on lifestyle and physical activity among adults with prehypertension or stage 1 hypertension resulted in significant reduction in cardiovascular risk (12% to 14% relative reduction in the 10-year Framingham Coronary Heart Disease Risk Score), which was maintained at 18 months.^21^

The present IMPACT-RMD project has a great advantage relative to being a typical, academically-led research study; that the highest authority for RMDs in Europe -the EULAR and its Educational Team - will lead on the implementation of the project and outreach to RMD doctors and healthcare professionals in European countries. This has the potential to be a sustainable approach for embedding physical activity in clinical care of RMDs, an intervention with multiple demonstrated benefits on different RMD outcomes.
